# The nuclear retinoid‐related orphan receptor ROR *α* controls circadian thermogenic programming in white fat depots

**DOI:** 10.14814/phy2.13678

**Published:** 2018-04-19

**Authors:** Chloé Monnier, Martine Auclair, Gala Le Cam, Marie‐Pauline Garcia, Bénédicte Antoine

**Affiliations:** ^1^ INSERM CNRS Centre de Recherches St‐Antoine (CRSA) Sorbonne Université Paris France

**Keywords:** Browning, circadian control, ROR*α*, WAT

## Abstract

The ROR *α*‐deficient staggerer (sg/sg) mouse is lean and resistant to diet‐induced obesity. Its thermogenic activity was shown to be increased not only in brown adipose tissue (BAT), but also in subcutaneous white adipose tissue (WAT) where UCP1 content was enhanced, however, without *Prdm16* coexpression. Our observation of partial multilocular lipid morphology of WAT in sg/sg mice both in the inguinal and perigonadal sites led us to focus on the phenotype of both fat depots. Because ROR *α* is a nuclear factor acting in the clock machinery, we looked at the circadian expression profile of genes involved in thermogenesis and browning in WAT and BAT depots of sg/sg and WT mice, through real‐time quantitative PCR and western blotting. This 24‐h period approach revealed both a rhythmic expression of thermogenic genes in WAT and an increased browning of all the WAT depots tested in sg/sg mice that indeed involved the canonical browning process (through induction of *Pgc‐1α* and *Prdm16*). This was associated with an enhanced isoproterenol‐induced oxygen consumption rate of WAT explants from sg/sg mice, which was reproducible in WT explants by treatment with a ROR *α* inverse agonist SR 3335, that induced a parallel increase in the UCP1 protein. Inhibitors of browning differentiation, such as TLE3 and RIP140, could be new targets of ROR *α* that would be rather implicated in the whitening of adipocytes. Our study showed the pivotal role of ROR *α* as an inhibitor of the thermogenic program in WAT, the role that could be counteracted in vivo with the ROR *α* antagonists currently in development.

## Introduction

Brown adipocytes are thermogenic cells predominantly found in brown adipose tissue (BAT). However, brown adipocytes can also be found in white adipose tissue (WAT) (e.g., inguinal WAT) following adrenergic stimulation (Ghorbani and Himms‐Hagen 1997). The induction of the thermogenic program of WAT, a process named browning (resulting in beige WAT), is thought to be a potential way for treatment of obesity and its related metabolic disturbances (Dulloo 2011). Indeed, pathological expansion of adipose tissues is the hallmark of obesity, and thermogenesis burns fatty acids to produce heat, which provides an outlet for fat stores. The discovery of new molecular mechanisms of browning is thus fundamental in order to understand this process that favors the elimination of fatty acids rather than their excessive storage, storage which in turn leads to adipocyte hypertrophy and subsequent tissue dysfunctions observed in obese states.

The nuclear receptor (NR) superfamily coordinates diverse aspects of organ physiology. Members of the NR family are abundantly expressed in tissues with major metabolic activity such as adipose tissues, liver, and skeletal muscle. By sensing hormones, vitamins, and dietary lipids, NRs coordinate molecular genetic programs that regulate lipid and carbohydrate metabolism (Chawla et al. 2001). In vivo studies have revealed that some of the orphan NR regulates the pathophysiology of obesity and insulin resistance (for a review see Pearen and Muscat (2012)). The orphan nuclear factor retinoid‐related orphan receptor *α* (ROR*α*), for example, is involved in several processes that are deregulated in pathologically expanding adipose tissue such as inflammation, adipogenesis, circadian rhythms, and lipid homeostasis (see Cook et al. 2015 for a review).

The ROR*α* loss‐of‐function mutant mouse – the Staggerer (sg/sg) mouse – harbors an interesting lean phenotype despite hyperphagia, with fat mass decreased by half when compared to its wild‐type (WT) littermates. Moreover, the small size of their adipocytes is not due to a defect in adipogenesis, which is indeed improved (Duez et al. 2009); their adipocytes are highly sensitive to insulin action and are able to increase their lipid storage capacity in response to a high‐fat (HF) diet (Kadiri et al. 2017). These mice are protected against HF diet‐induced obesity (Lau et al. 2008) and related systemic insulin resistance (Kang et al. 2011), without harboring any liver steatosis or hyperlipidemia. Such characteristics and the fact that the physical activity of sg/sg mice is less than that of WT littermates (Akashi and Takumi 2005) begs the question of where the energy derived from the food intake is disposed of.

The higher metabolic rate of sg/sg mice was first demonstrated on chow diet (Bertin et al. 1991), then confirmed in HF‐fed sg/sg mice (Kang et al. 2011) where the higher rate of energy expenditure was partly attributed to an increase in heat generation associated with an increase in uncoupling protein 1 (UCP1) expression in BAT. UCP1 is critical for nonshivering heat production (Cannon and Nedergaard 2011). More recently, the reduced adiposity of sg/sg mice was also credited to the browning of one of its WAT depot, the inguinal subcutaneous adipose tissue (IAT), coupled with the increased *Ucp1* gene and protein expression in IAT, which was interestingly not observed in epididymal adipose tissue (EAT) (Lau et al. 2015). Furthermore, this brown WAT activation was observed to be independent of PRDM16, and proposed as an alternative method of browning, although PRDM16 was shown to be a determinant for a brown fat‐like program in IAT (Seale et al. 2011).

Observing the partial multilocular lipid droplet morphology of WAT in ROR*α* sg/sg mice, both at the inguinal and epididymal sites, we investigated, in this study, some thermogenic genes and proteins expression profiles over a 24‐h period in order to evaluate the possibility of any circadian variation of the brown phenotype in IAT and EAT of sg/sg and WT mice. Indeed, *Ucp1* gene expression exhibits a strong circadian rhythmicity in BAT, in which temperature is high during the active phase (dark phase) and low in the inactive phase (light phase) of mice (Gerhart‐Hines et al. 2013). This raised the possibility of a cyclic expression of *Ucp1* in WAT that could have been missed previously since investigations usually performed on mice are done at the beginning of the light phase (i.e., in their inactive period). Moreover, the circadian rhythmicity of *Ucp1* gene expression in BAT was shown to be controlled by Rev‐erb*α*, (Gerhart‐Hines et al. 2013), a NR that shares with ROR*α* many of its molecular targets and also belongs to the clock machinery (Cook et al. 2015). Indeed, the diurnal oscillations in behavioral activity and physiology are tightly controlled by the circadian clock (Bass and Takahashi 2010).

We chose to study key proteins involved in thermogenesis and browning, such as the mitochondrial protein CPTIβ which is involved in mitochondrial transportation of acyl‐CoA during fatty acid assimilation, COX_4,_ implicated in oxidative phosphorylation, CIDEA which is associated with the regulation of lipid droplet size, and DIO_2_, an enzyme converting thyroid hormone T4 into bioactive T3. We also studied transcriptional activators and repressors of browning: EHMT1, PRDM16, PGC‐1*α*, PPAR*α*, ERR*α*, TLE3, and RIP140, respectively, which control *Ucp1* gene transcription, as review in (Bonet et al. 2013).

## Material and Methods

### Animals and tissues

sg/sg mice (a spontaneous mutation in C57BL/6 strain) were obtained by crossing heterozygous sg/+ mice, given by Pr. J. Mariani’ s laboratory. All animals’ care and use procedures were in accordance with the guidelines of the Charles Darwin Ethics Committee (Ce5/2010/034). Mice were housed in a specific pathogen‐free environment in a temperature‐controlled room maintained at 24°C, with a 12 h light/dark cycle (lights on from 8 h to 20 h). Water and food (A03, UAR, Epinay‐sur‐Orge, France) were provided ad libitum. Male mice, aged 16–26 weeks, were used in this study except when mentioned. Mice were sacrificed by cervical dislocation at 4 h intervals over a 24‐h period with four to six mice at each time point. Perigonadal and inguinal (white), and interscapular (brown) adipose tissues were dissected, snap‐frozen in liquid nitrogen and stored at −80°C before RNA extraction or protein homogenate preparation. For histological examination, WAT was fixed in formalin. For functional studies, adipose tissues were minced into small pieces (5–10 mg) and incubated in DMEM containing 10 mmol/L glucose and 2% bovine serum albumin (BSA) before OCR measurement or adipocyte isolation.

### Mitochondrial mass estimation

Fat pads were excised and adipocytes were isolated from the stromal vascular fraction after collagenase digestion (Liberase TM, Roche) as previously described (Dusaulcy et al. 2011). Mitochondrial mass was measured by the fluorescent labeling of the Mito Tracker Red probe (MTR, M‐7512, molecular Probes, Eugene, OR) in isolated adipocytes from IAT and EAT of male mice and normalized to the protein content.

### RNA extraction, cDNA synthesis and quantitative PCR

Adipose tissue mRNA was extracted using the RNeasy Lipid Tissue Mini Kit (Qiagen, Courtaboeuf, France), then reverse‐transcribed using the High‐Capacity cDNA Reverse Transcription Kit (Applied Biosystems Carlsbad, CA, USA). Quantitative PCR of the genes of interest was performed using a SYBR Green and a Light Cycler 480 Real‐Time PCR System (Roche Diagnostics, Meylan, France) and the specific primers listed as followed: for *Cpt1b*, ATCATGTATCGCCGCAAACT (forward) and CCATCTGGTAGGAGCACATGG (reverse); for *Pparα*, GCACTGGAACTGGATGACAG (forward) and TTTAGAAGGCCAGGACGATCT (reverse); for *Pgc‐1α*, CAACCGCAGTCGCAACAT (forward) and TGGGAACCCTTGGGGTCA (reverse); for *Ucp1*, ACAGAAGGATTGCCGAAAC (forward) and AGCTGATTTGCCTCTGAATG (reverse); for *Dio2*, CCTCCTCGATGCCTACAAAC (forward) and GCTGGCAAAGTCAAGAAGGT (reverse); for *Cidea*, GATAGGGCAGTGATTTAAGA (forward) and GGTCAGTAGGACCTTCTTAGT (reverse); for *Prdm16*, ATGTGCTTAATTCCACCTTA (forward) and GGAGAGGAGTGTCTTCAGAG (reverse); for EHMT1, GGCACCTTTGTCTGCGAATAC (forward) and AGAACCGAGCGTCAATGCAG (reverse); for TLE3, TGGTGAGCTTTGGAGCTGTT (forward) and CGGTTTCCCTCCAGGAAT (reverse); and for *Gapdh*, CAAGGAGTAAGAAACCCTGGACC (forward) and CGAGTTGGGATAGGGCCTCT (reverse). Gene expression was normalized to GAPDH and data analysis was based on the ΔΔCt method.

### Protein extraction and western blot analysis

Frozen adipose tissue was homogenized on ice in tissue protein lysis buffer (Euromedex, Souffelweyersheim, France) then centrifuged first at 14,000 *g*, 4°C for 8 min to remove lipids, and then at 20,000 *g*, 4°C for 10 min to remove the insoluble material. Supernatants were subjected to SDS–PAGE and western blotted with antibodies against PGC‐1α (sc‐5816) (Santa‐Cruz Biotechnology, Heidelberg, Germany) and against PRDM16 (ab 106410), PPARα (ab 8934), UCP1 (ab 10938), and HSL (ab 45422), (Abcam, Paris, France).

### Isoproterenol‐induced oxygen consumption rate of adipose tissue explants

Tissues were minced into 5–10 mg fragments and similar amounts of explants were distributed in wells of a microplate with an oxygen sensor (Oxoplate, Presens, Regensburg, Germany) in DMEM without phenol red, supplemented with 0.5% BSA (*n* = 8 by explants) and incubated at 37°C for at least 30 min to homogenize their temperature. Then, prewarmed 1 M isoproterenol was added to four of the wells, the plate was closed with strips of adhesive foil, and incubated in the microplate reader Fluoroskan Ascent (Thermo Fisher Scientific, Dreieich, Germany) for 1 h at 37°C. The fluorescent signals were measured for 40 min with a measurement interval of 5 min, using the filter combinations 544/650 nm for the indicator dye and 544/590 nm for the reference dye. The results from four wells of each condition was averaged and normalized to total mg tissue.

### ROR*α* chemical ligand

SR3338 was purchased from Cayman (Interchim, Montigny le Bretonneux, France). Resuspended in DMSO, it was used at 10 *μ*m.

### Statistical analysis

Values are presented as means ± SEM. Statistical analysis was performed by a one‐way or two‐way ANOVA or by unpaired Student‘s *t*‐test as mentioned in the figures caption. *P* < 0.05 was considered the limit for statistical significance.

## Results

### Lean and insulin‐sensitive phenotype of sg/sg mice regardless of the diet

Six‐month‐old sg/sg mice weighed 20% less and had a lower body mass index (BMI) than their WT littermates when both were fed a standard diet (SD) (Fig. S1A), even though their food intake was twofold higher (Fig. S1C). Sg/sg mice had a lower HOMA‐IR index and exhibited a fat mass reduced by half, with adipocyte size being four times smaller than in WT littermates. This concerned all white fat depots, although with a preferential loss of visceral fat depots (Kadiri et al. 2017). Glucose tolerance and insulin sensitivity were not different from WT mice in male sg/sg mice that were SD fed (Fig. S1B), as found by Kang et al. (2011), but it was improved in female sg/sg (not shown), as reported by Lau in male, however (Lau et al. 2011).

When sg/sg and WT mice were fed a Western diet (WD) for 16 weeks, they gained 2 g and 5 g on average, respectively, but only the WT mice had a diet‐induced increase in BMI, glycemia and insulinemia resulting in an increased HOMA‐IR (Fig. S1A). This was associated with an increase in WAT mass of about fourfold in WT mice and twofold in sg/sg mice (Kadiri et al. 2017). WD‐fed sg/sg mice conserved a higher tolerance to glucose than WD‐fed WT mice and did not acquire any resistance to insulin at the systemic level (Fig. S1B), exhibiting even improved insulin sensitivity at the level of WAT depots (Kadiri et al. 2017).

**Figure 1:**
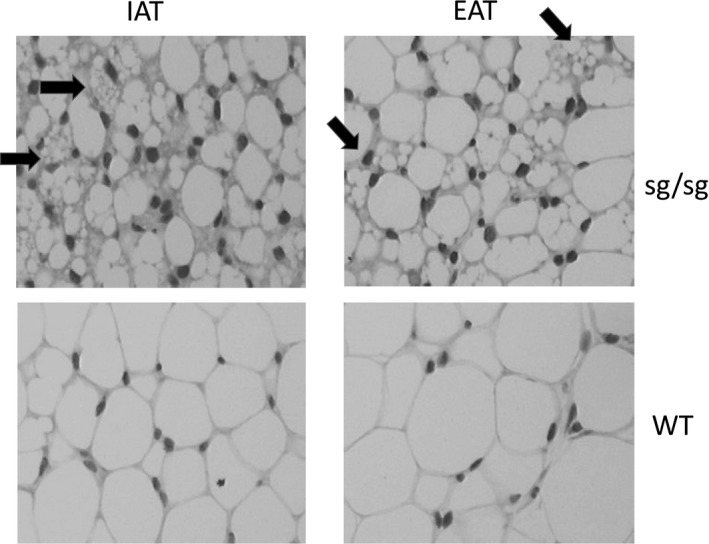
Histology of inguinal (IAT) and epididymal (EAT) adipose tissue of 26‐week‐old male sg/sg and WT mice. Staining with hematoxylin and eosin. Arrows point out some brown‐like adipocytes with multilocular lipid droplet morphology.

At the adipocyte level, we were interested in the multilocular lipid droplet morphology of WAT in sg/sg mice, both at the inguinal and epididymal sites, as shown in Figure 1A. This could be related to the “browning” of several WAT depots in these mice, which we chose to investigate over a 24‐h period.

### Circadian rhythmicity of “browning” genes expression in inguinal and epididymal WAT and BAT of sg/sg and WT mice

**Figure 2:**
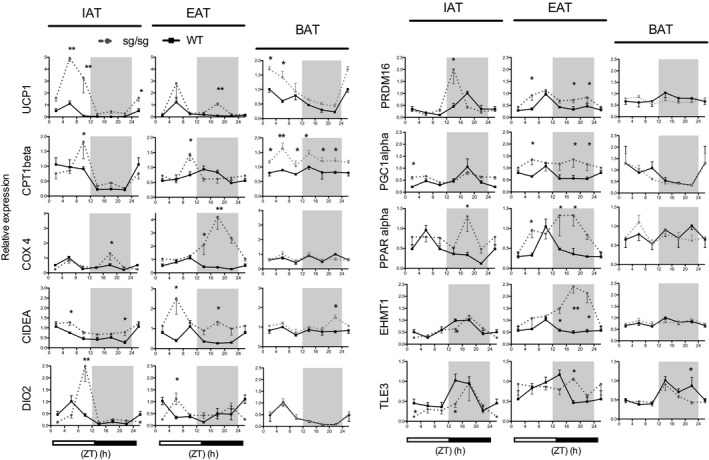
Circadian pattern of thermogenic genes expression in two WAT depots and BAT of sg/sg and WT mice. Male sg/sg and WT mice were sacrificed at 4 h intervals over a 24‐h period from ZT2 to ZT22 (ZT0 is the time of lights on, ZT12 is the time of lights off). For each gene, the period with the highest level in WT mice was set to 1. *N* = 3–6 per group. Data are mean ± SEM versus WT mice. *P < 0.05, **P < 0.01 by two‐way ANOVA with Bonferroni test.

Considering that ROR*α* is an actor of the core clock machinery, we suspected that its absence in sg/sg mice could have an impact not only on target genes transcription but also potentially on the rhythmicity of thermogenic gene expression in WAT. We compared the quantitative expression pattern of several mRNAs, coding for enzymes and transcription factors involved in thermogenesis and browning over a 24‐h period, in WAT (IAT and EAT) and BAT of sg/sg mice and WT littermates (Fig. 2). ZT0 is the beginning of the light phase and ZT12 is the beginning of the dark phase over a 24‐h period.

First, we focused on UCP1 as it allows heat production in BAT (Nichols 1976) in response to adrenergic stimulation (nonshivering thermogenesis). We first confirmed that *Ucp1* gene expression exhibits a strong circadian rhythmicity in BAT of WT mice (Gerhart‐Hines et al. 2013), with a peak at ZT2 and a nadir (lowest level or trough) at ZT 18‐22. We then showed that this rhythmicity was conserved in sg/sg mice although with a higher amplitude (Fig. 2). In both IAT and EAT of WT mice, our data illustrated a diurnal rhythm of *Ucp1* gene expression, with a peak around ZT6. In sg/sg mice, the amplitude of this peak was highly increased (absolute level multiplied by five) in IAT, but not in EAT, confirming the data of Lau et al. (2015) that were probably obtained around ZT6‐8. However, our approach allowed the observation of a second peak of *Ucp1* mRNA in EAT of sg/sg mice during the night (Fig. 2). When the area under the curves (AUC) were calculated, our data confirmed a sixfold and a 2.4‐fold increase in *Ucp1* mRNA amount over a 24‐h period in IAT and EAT of sg/sg mice compared to that of WT mice, respectively.

We then compared the circadian profiles of expression of other genes that are customarily involved in thermogenesis between sg/sg and WT mice.

First, considering the BAT of WT mice*,* only five of the 12 others genes examined exhibited a circadian rhythmicity (*Dio2*,* Pgc‐1α*,* Tle3*,* Rip140*,* and Rev‐erbα)* (Fig. 2 *and* Fig. S4 *)*. The decreased expression of *Ucp1* during the night could thus be linked to the simultaneous decrease in two activators (*Pgc‐1α* and *Dio2*) combined with an increase in an inhibitor (*Tle3)*. The overexpression of *Ucp1* in the BAT of sg/sg mice did not seem to be related with any variation in the rhythmicity or expression level of these genes, excepted a parallel increase in *Cpt1β* that seemed to be expressed two times more than in WT mice.

Considering the WAT in WT mice, some transcripts exhibited more circadian variations than in BAT (*Cpt1β*,* Prdm16*,* Pparα*,* Ehmt1*, and *Errα*). This phenomenon was amplified in the WAT of sg/sg mice where almost all the transcripts studied exhibited circadian variations, which suggests a higher impact of the loss of ROR*α* on the circadian rhythmicity in the white lineage when compared to the brown lineage.

We delineated some different patterns between fat depots in one same genotype and between mice genotypes (Fig. 2). Although the *Cpt1β* or *Dio*_*2*_ transcripts peaked more acutely in sg/sg mice around ZT9 in both fat depots, their global circadian rhythm of expression looked similar to that of their respective patterns in WT mice. On the contrary, *Cox4*,* Pparα, Errα*, and *Ehtm1* transcripts peaked during the dark period in sg/sg mice, whereas they peaked during the light period in WT mice. Additionally, our horizontal approach allowed to reveal the increased expression of *Prdm16* in both IAT and EAT depots of sg/sg compared to that of WT mice; the peak of *Prdm16* mRNA being located in the dark period in IAT of mutant mice and thus not detectable in the light period.

Overall, when we compared the AUC of gene expression profiles in each genotype, our data showed that the mRNA amount over a 24‐h period was significantly increased in sg/sg mice when compared to WT for *Ucp1*,* Cidea*,* Dio2*,* Prdm16*, and *Pparα* in IAT and, for *Cox4*,* Cidea*,* Prdm16*,* Pgc‐1α*,* Pparα, Ehtm1*,* and Errα* in EAT. This suggested that not only the IAT but also the EAT is browning in RORα‐deficient mice, although to different degrees. Indeed, IAT is at least 10‐fold more enriched in *Ucp1* and *Cpt1β* mRNAs than EAT in sg/sg mice; however, it is the opposite for *Cox4* that is eightfold more enriched in EAT.

### Circadian rhythmicity of “browning” protein amounts in inguinal and epididymal WAT in sg/sg *and* WT mice

**Figure 3:**
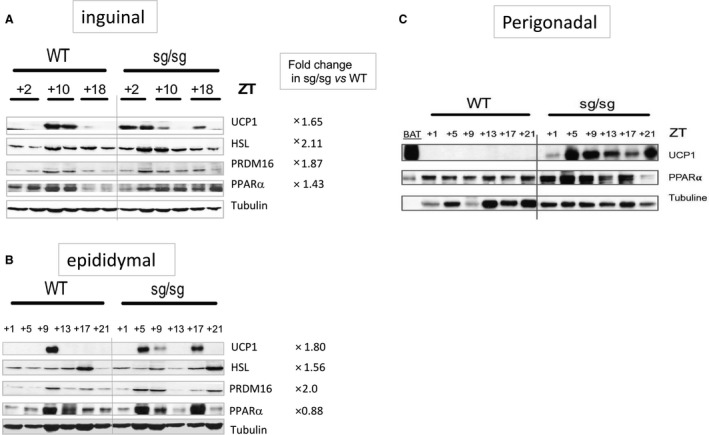
Circadian pattern of thermogenic protein expression in two WAT depots of sg/sg and WT mice. Western blot analysis of WAT protein lysates over a 24‐h period. Protein extracts from two mice at each indicated point were pooled in B. (A) and (B) concerned IAT and EAT from male mice, respectively, and (C) concerned perigonadal AT from female mice. Fold change referred to the sum of densitometric signals compared to a tubulin loading control over the 24‐h period in each genotype.

Because mRNA amounts do not always reflect protein amounts, we performed western blots to confirm the presence and the rhythmicity of expression of thermogenic protein in the WAT depots of sg/sg mice. As for mRNAs, we investigated their amount in the IAT (Fig. 3A) and EAT (Fig. 3B) of male mice sacrificed at various ZT over a 24‐h period.

Our data showed an expression of UCP1 protein restricted around ZT10 in both fat depots of WT mice, subsequent to the mRNA peak at ZT6 observed in Figure 2. In sg/sg mice, UCP1 is almost twice more frequently observed in both fat depots over a 24‐h period compared to WT mice; one can take note of its presence earlier, at ZT2 for IAT and ZT5 for EAT, and later at ZT18 for both fat depots. These results fit well with the mRNAs profiles obtained in Figure 2 and thus confirmed the higher UCP1 content in both fat depots of sg/sg compared to WT mice.

The circadian expression of HSL in WAT depots of WT mice was confirmed (Shostak et al. 2013) and peaks were found to occur relatively earlier in IAT (or later in EAT) of sg/sg mice with an overall twofold enriched expression, when compared to WT. The same observation was made for PRDM16 protein that appeared about twice more present over a 24‐h period in both WAT depots of sg/sg mice than of WT and which distribution fitted with UCP1 content. PPARα was present all over the 24‐h period in IAT of sg/sg mice while its content decreased during the night in WT mice. In EAT of sg/sg mice, its content corresponded to the presence of UCP1.

We wondered about the relatively higher loss of perigonadal fat in the female compared to that observed in male sg/sg; therefore, we also analyzed its potential browning in this gender. Figure 3C demonstrated a substantial content of UCP1 in this fat depot in female sg/sg mice, content that is even more important than in their respective IAT (not shown). It was also accompanied by an enrichment in PPAR*α*.

We also compared the UCP1 protein content in the perigonadal fat of male and female mice that have been WD fed for 16 weeks and sacrificed at ZT2. Data from Figure S2B confirmed a higher content of UCP1 in perigonadal AT of sg/sg mice from both gender by comparison with WT mice. This UCP1 enrichment was associated with a respective increased content of PRM16, PGC‐1α, and PPARα. This data suggested that the browning of perigodanal fat depot could participate to the obesity‐resistant phenotype of sg/sg mice fed a WD. Indeed, the diet‐dependent increase in UCP1 content was in the range of 1.5‐ and 2‐fold for WT and sg/sg mice, respectively (not shown).

Finally, Figure S2A showed the condensation occurring in a metabolic cage accommodating a WD‐fed male sg/sg mouse, compared to a WD‐fed male WT mouse, confirming the higher heat production of mutant mice.

### In vitro respiration of BAT and WAT of sg/sg *and* WT mice

**Figure 4:**
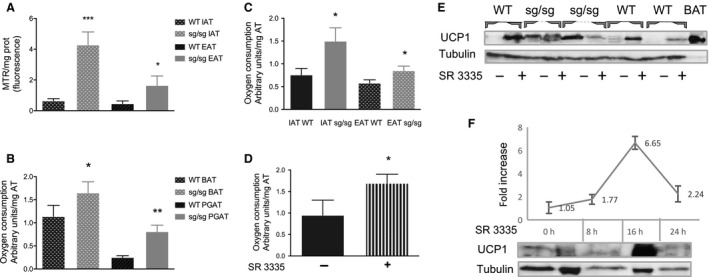
*In vitro* respiration of BAT and WAT of sg/sg versus WT mice. (A) Mitochondrial mass was measured by the fluorescent labeling of the MTR probe in isolated adipocytes from IAT and EAT of male mice and normalized to the protein content. *N* = 5. Data are mean ± SEM versus WT mice. *P < 0.05, ***P < 0.005 by Student's *t*‐test. (B, C, D) Isoproterenol‐induced oxygen consumption rate of adipose tissue explants. Tissues were minced into 5–10 mg fragments and similar amounts of explants were distributed in wells of a microplate with an oxygen sensor (Oxoplate, Presens, Regensburg, Germany) and incubated with or without IPR for 30 min. *P < 0.05, **P < 0.01 by Student's *t*‐test. (B) BAT and perigonadal WAT from female mice. *N* = 7. (C) IAT and EAT from male mice with similar body weights. *N* = 4. (D) WAT from male WT mice ex vivo preincubated for 20 h with 10 *μ*m SR 3335, an inverse agonist of ROR ***α*** (*N* = 4). (E) Western blot analysis of protein lysates from IAT of WT and sg/sg mice treated for 20 h with 10 *μ*m SR 3335 or DMSO. (F) Induction kinetic of UCP1 protein in IAT of WT mice**.** Explants (from the same mouse) have been treated with 10 *μ*m SR 3335 for the indicated periods. One “zero time” was frozen at ZT2 (corresponding to the 16‐h incubation period) and the other at ZT10 (corresponding to the 8‐ or 24‐h incubation period) in order to remedy any endogenous circadian fluctuation of UCP1 protein. The curve illustrated both the appearance and the disappearance of the UCP1 protein, whose half‐life seems to be inferior to 24 h in such “ex vivo” procedure.

Mitochondrial mass was measured by fluorescent labeling of the MTR probe in isolated adipocytes from IAT and EAT of male mice and normalized to the protein content. Figure 4A shows an enrichment in mitochondria of both WAT depots in sg/sg compared to WT mice, by 6.6‐fold in IAT and by 3.6‐fold in EAT.

To get an idea about the thermogenic capacity of these fat depots, we measured the isoproterenol‐induced oxygen consumption rate (OCR) of explants in vitro by using a microplate fitted with an oxygen sensor. We first tested the procedure with thermogenic‐positive explants (BAT samples) that we compared to perigonadal WAT (PGAT) from female at ZT5. Figure 4B shows the confirmation that more oxygen was consumed by BAT from sg/sg mice than WT, as shown by Lau et al. (2015) and demonstrated for the first time that the OCR of PGAT was increased threefold in sg/sg compared to WT mice. We then performed the same experiment in male mice at ZT10 to fit the peak of UCP1 protein expression (Fig. 4C). The OCR of both WAT depots was increased in male sg/sg compared to WT mice (2‐fold and 1.5‐fold in IAT and EAT, respectively).

Finally, to assess whether we could replicate ex vivo the increased OCR of WAT from sg/sg mice, we treated IAT explants from WT mice with a ROR*α* inverse agonist (SR 3335) (Kumar et al. 2011) for 20 h and then tested the OCR at ZT0. Figure 4D illustrated the capacity of SR 3335 to increase the OCR of IAT from WT mice, thus suggesting the role of ROR*α* as a pivotal inhibitor of browning. To further link the observed increased OCR to UCP1 amount, we measured the effect of SR 3335 on UCP1 protein content. Data from Figure 4E clearly demonstrated that antagonizing ROR*α* resulted in the appearance of UCP1 protein in IAT from three different WT mice, whereas that effect was not observed in IAT from sg/sg mice. We also checked the peak of UCP1 protein induction after antagonizing ROR*α* by SR 3335 and data from Figure 4F suggested de novo protein synthesis rising 16 h after treatment before decreasing again.

## Discussion

This study confirms the role of ROR*α* in the thermogenic program of WAT, but unlike previous data (Lau et al. 2015), we show here that it also concerns the visceral depot, and not only the subcutaneous one. We also show that it involves the canonical browning process, through the induction of *Pgc‐1α* and *Prdm16*. This was done by characterizing a circadian control of brown‐associated transcripts and proteins in different WAT depots of ROR*α*‐deficient sg/sg and WT mice. Browning of WAT was functionally assessed by the observation of an increased OCR in response to isoproterenol. While this browning was higher in IAT than in EAT in male, we discovered a substantial UCP1 enrichment in perigonadal WAT of female sg/sg mice. This suggests an inhibitory role of ROR*α* on browning, the role that is more pronounced in the visceral WAT of females than in that of males. Considering that visceral fat depot hypertrophy is linked to the occurrence of metabolic syndrome and that antagonists of ROR*α* are in development (some of them with in vivo activity) (Kojetin and Burris 2014), our data opens a new field of potential targets for antiobesity drugs. Indeed, we observed that the increased OCR and UCP1 protein content of WAT in sg/sg mice is reproducible in vitro by incubating adipose tissue explants of WT mice with a ROR*α* inverse agonist.

There is a circadian rhythm in nutrient metabolism that primes the organism to assimilate or mobilize nutrients at specific times of the 24‐h daily cycle (Panda 2016). There are many circadian‐controlled gene oscillations in adipose tissues (Zvonic et al. 2006), such as thermogenic gene programming in BAT. *Ucp1* expression exhibits a strong circadian rhythmicity, being (among others) controlled by Rev‐erb*α*, a powerfull circadian transcriptional repressor (Gerhart‐Hines et al. 2013). In this context, and because ROR*α* is also a partner of the clock machinery, we investigated the possibility of a circadian control of thermogenic genes in WAT, which we then compared to the pattern in BAT, in view of offering an explanation for how ROR*α*‐deficient mice could be resistant to obesity.

In the BAT of WT mice*,* only half of the transcripts examined exhibited a circadian rhythmicity (*Ucp1*,* Dio2, Pgc1a, Tle3*,* and Rip140)*. *Ucp1* gene expression decreased coincidentally to both the decrease in two transactivators (*Pgc1α* and *Dio2*) and the increase in two inhibitors (*Rev‐erbα* and *Tle3)*. Such data confirm the circadian control of *Ucp1* gene expression and suggest a protein half‐life minor than 24 h to translate this circadian thermogenic plasticity (Gerhart‐Hines et al. 2013). Indeed, we observed a protein peak 16 h after treatment with SR 3335, and protein content was greatly reduced 8 h later, that is, 24 h after induction.

In the BAT of sg/sg mice, the rhythmic expression of *Ucp1* transcript was similar to that in WT, but with a higher amplitude, possibly related to a weak decrease in *Tle3*, a white‐selective cofactor that counters PRDM16 activity (Villanueva et al. 2013). The only other difference we identified between the BAT of the two genotypes concerned *CPT1*b that was expressed about two times more in sg/sg than in WT mice, as previously shown (Lau et al. 2015).

Concerning the WAT of WT mice, the peak of *Ucp1* mRNA occurred at the same period in the two depots (ZT6), perigonadal and inguinal, thus being shifted to a later peak of expression than in the BAT (ZT2) (Fig. 2 and Gerhart‐Hines et al. 2013). Contrary to that observed in BAT, it was associated with circadian modulations of all the tested regulators of *Ucp1* transcription. This is particularly the case for *Pparα* transcripts that were also found to be different between WAT and BAT (Yang et al. 2006).

In the WAT of sg/sg mice, the major peaks of *Ucp1* expression happen at similar times to that of WT mice; however, they display more amplitude, in relation with higher expression of effectors, such as *Dio2, Prdm16*,* Pgc1α*, and *Pparα*, which also exhibited more pronounced circadian variations than in WT mice. This approach allowed us to confirm the browning of IAT of sg/sg mice, mentioned in (Lau et al. 2015), but additionally to show that it indeed takes place because of the sur‐expression of several classical brown regulators**,** *Prdm16* in particular, which increased expression was only noticeable during the dark phase (and thus not seen in Lau et al. (2015)).

The screening of these brown markers over a 24‐h period allowed to discover that the browning process also concerned the perigonadal depot of sg/sg mice, that is, to say several WAT depots, with a striking gender difference observable in the female. In general, IAT is particularly prone to browning in mice exposed to cold and beta‐agonists (Seale et al. 2011), but this phenomenon is much less common in visceral fat depot. Our data showed a browning phenotype of the major fat depots of sg/sg mice compared to WT, including EAT. Such findings highlight the restricted information obtained when two genotypes were compared in the light (and inactive) period only, especially considering mice mutated for a clock‐machinery gene and/or a gene concerning metabolic pathways. In such studies, the mRNA analysis of EAT in sg/sg mice in the light period only would have led to the conclusion of no differences with WT mice because it would be missing the highest expression in the dark period.

The measure of the OCR of fat depots explants in response to isoproterenol strongly suggested some alternate functionality of this observed browning of WAT in the absence of ROR*α*. While the actual physiological importance of WAT thermogenesis is still under investigation, as discussed in (Keipert and Jastroch 2014), some data for a thermogenic function of beige adipocytes looks promising. For example, mice with BAT ablation exhibit WAT browning and compensatory ways of maintaining body temperature in the cold (Schulz et al. 2013). In the same way, we observed that, after being fed a WD‐diet, there was noticeable condensation in the metabolic cages of sg/sg mice (Fig. S2) whereas no increase in BAT mass was observed (not shown). In addition, Figure S3 well illustrated the diet effect on the emergence of multilocular lipid droplets in WAT and BAT depots of female sg/sg mice. This suggests that the increased thermogenesis occuring in WAT and BAT of sg/sg mice when being fed a WD helps for dissipating the energy surplus derived from the diet.

Increased levels of norepinephrine in BAT from sg/sg mice have been mentioned in Bertin et al. (1991), thus making it possible for the browning of the WAT to be a consequence of an activated adrenergic signaling in these mice. However, comparable increases of oxygen consumption in response to *β*3‐agonist in both genotypes were observed (Lau et al. 2015), as well as lower *β*3‐AR expression in WAT from sg/sg mice when compared to WT (Lau et al. 2008). Thus, the increased thermogenic activity in sg/sg mice was interpreted more as a cell autonomous effect than as being driven by the CNS. Given that ex vivo treatment of WAT from WT mice with a ROR*α* inverse agonist increased both UCP1 protein amount (and Cox 2 as well, not shown) and oxygen consumption rate also implies a cell autonomous effect and suggest this NR as a new potential target for inducing the browning program of WAT.

Browning is thought to be under genetic control because of genetic variations between different strains of mice (Guerra et al. 1998). From a molecular point of view, our data would suggest that thermogenesis in white adipocytes is inhibited by ROR*α*. Quantities of ROR*α* mRNA and protein both increase during white adipocyte differentiation (between J4 and J8) (Duez et al. 2009; Ohoka et al. 2009), thus possibly participating to the repression of a brown phenotype that was, however, not tested at the time of these experiments (in 2009). Indeed, our data show that the RORα absence in sg/sg mice induced a complete browning transition, illustrated by the simultaneous activation of several partners of the thermogenic program. In this case, it would undoubtedly be an indirect control since RORα is a transcriptional activator. Interestingly, in sg/sg mice, we also observed a decreased expression of two repressors of *Ucp1* and of the brown fat program in the IAT (*Tle3 and Rip140*), and the loss of cycling of *Tle3* transcripts associated with a significant increase in *Ehmt1* in the EAT. This suggests that *Tle3 and Rip140* could be direct targets of ROR*α* transcription.

ROR*α*, encoded by *NR1F1*, constitutively transactivates target genes by binding as a mono/homodimer to ROR response element sequences (RORE) containing an AGGTCA half‐site preceded by a 5′‐A/T‐rich flanking sequence in DNA promoter regions of target genes. Rev‐erb*α*, encoded by *NR1D1*, is able to bind the same DNA sequence as a homodimer (Moraitis and Giguere 1999) and acts as a competitor of ROR*α* DNA‐binding. Considering that Rev‐erb*α* is a suppressor of transcriptional activity, it represses ROR*α* target genes expression. Rev‐erb*α* was shown to bind to the *Ucp1* promoter (Gerhart‐Hines et al. 2013), thus confirming the existence of RORE in the *Ucp1* promoter. However, Rev‐erb*α* repressed *Ucp1* expression in BAT in response to cold (Gerhart‐Hines et al. 2013), implying that ROR*α* would be a potential transactivator of *Ucp1* expression, which is the opposite of what we have observed in our experiments.

However, there were divergences of opinion on the role of Rev‐erb*α* in thermogenesis because in vivo treatment of mice with Rev‐erb*α* agonist increased whole body energy expenditure (Solt et al. 2012). Those Rev‐erb*α* agonists theoretically lower ROR*α* target genes expression by stabilizing the interaction between Rev‐erbα and the corepressor complex NcoR/HDAC3. We can therefore also advance the hypothesis that the increase in *Ucp1* expression in sg/sg mice could be due to the decrease in mRNA levels of Rev‐erb*α* (Fig. S4). Indeed, Rev‐erb*α* is a target of ROR*α*. In this scenario, *Ucp1* expression would increase in the WAT of sg/sg mice resulting from a decreased inhibition by Rev‐erb*α*. However, Rev‐erb KO mice exhibit an obese phenotype, and another argument against this theory is the fact that the perigonadal adipose tissue of female sg/sg mice expressed increased levels of both *Ucp1* and *Rev‐erbα* mRNAs (not shown). This would mean that the action of ROR*α* on thermogenesis probably involves an outside molecular element.

Another way of explaining the unlikely action of ROR*α* on browning and thermogenesis programming lies in the RORE sequences found in promoter regions of target genes. The RORE site is a half site of the NR‐palindrome DNA‐binding site and possibly interacts with the binding of others NR. For example, it was shown that ROR*α* binding on the Perilipin gene (*Plin*) overlaps a peroxisome proliferator response element (PPRE) and prevent its activation by PPAR*γ* (Ohoka et al. 2009). Interestingly, the *Ucp1* promoter features an estrogen receptor‐related element (ERRE), which is very important for its transcriptional regulation (Debevec et al. 2007). Estrogen‐related receptor alpha (ERR*α*) is an orphan receptor known to play a central role in the control of energy homeostasis, and is also an important regulator of the mammalian circadian clock (Dufour et al. 2011). It is conceivable that the binding of ERR*α* could be prevented by the binding of ROR*α*. This would explain why the absence of ROR*α* causes an increase in *Ucp1* expression. We have thought about ERR*α* for this putative model because of the observed higher level of expression of *Ucp1* in female perigonadal adipose tissue compared to male adipose tissue. Indeed, ER*α* and ERR*α* have been found to regulate many of the same genes. Furthermore, ERR*α* appears to modulate the activity of ER*α* in various tissues including breast, uterus, and bone.

Finally, it was also shown that ROR*α* binding to Wnt/*β*‐catenin‐targeted promoters mediates transcription suppression, thus identifying RORa as reducing the Wnt/*β*‐catenin signaling and a potential antitumoral target (Lee et al. 2014). Consequently, activation of the canonical Wnt signaling influences the fate of many cell types and particularly inhibits differentiation of in vitro models of white and brown adipogenesis. Indeed, Kang et al. (2005) showed that activation of canonical Wnt signaling early in differentiation blocks brown adipogenesis, whereas activating Wnt signaling in mature brown adipocytes stimulates their conversion to white adipocytes, making Wnt a pivotal actor linking both brown and white program. Moreover, they observed in mice overexpressing Wnt 10B (FABP4‐Wnt 10b mice), that not only BAT development was impaired, but also WAT was resistant to browning under CL316,243 treatment. In this hypothesis, the absence of ROR*α* could enhance the Wnt signaling that can regulate adipogenesis, at various stage, from the commitment of preadipocytes to the brown differentiation phase (Tseng et al. 2010).

To conclude, our work has highlighted the role of ROR*α* in the modulation of browning and thermogenesis in WAT depots in mice. Interestingly, although ROR*α* is a transactivator of gene expression, our data suggested that it inhibits the thermogenic program of WAT. Although more work is required to tease out the exact molecular processes, this study demonstrates the pivotal role of ROR*α* in energy homeostasis and makes it an interesting therapeutic target for treatments focusing on artificially increasing energy expenditure as a way of handling obesity, all the more so since it has been shown that ROR*α* inverse agonists are also able to decrease hepatic neoglucogenesis (Kumar et al. 2011; Kadiri et al. 2015).

## Conflict of Interest

The authors have no conflict of interest to declare.

## Data Accessibility

## Supporting information




**Figure S1:** sg/sg mice are resistant to WD‐induced glucose intolerance and insulin resistance.

**Figure S2:** WD‐induced thermogenesis in sg/sg mice.

**Figure S3:** Histology of inguinal (IAT), perigonadal (PGAT), and brown (BAT) adipose tissue of 26‐week‐old female WT and sg/sg mice fed standard or Western diet.

**Figure S4:** Circadian pattern of some genes expression into the two WAT depots and BAT of sg/sg and WT mice.
