# Supplementary material for: The nuclear retinoid‐related orphan receptor ROR α controls circadian thermogenic programming in white fat depots
Source: Physiol Rep. 2018 Apr 19;6(8):e13678. doi: 10.14814/phy2.13678 (PMC5907938; doi:10.14814/phy2.13678)
Supplement: Supplementary file 1 — Figure S1: sg/sg mice are resistant to WD‐induced glucose intolerance and insulin resistance. [file PHY2-6-e13678-s001.pdf]

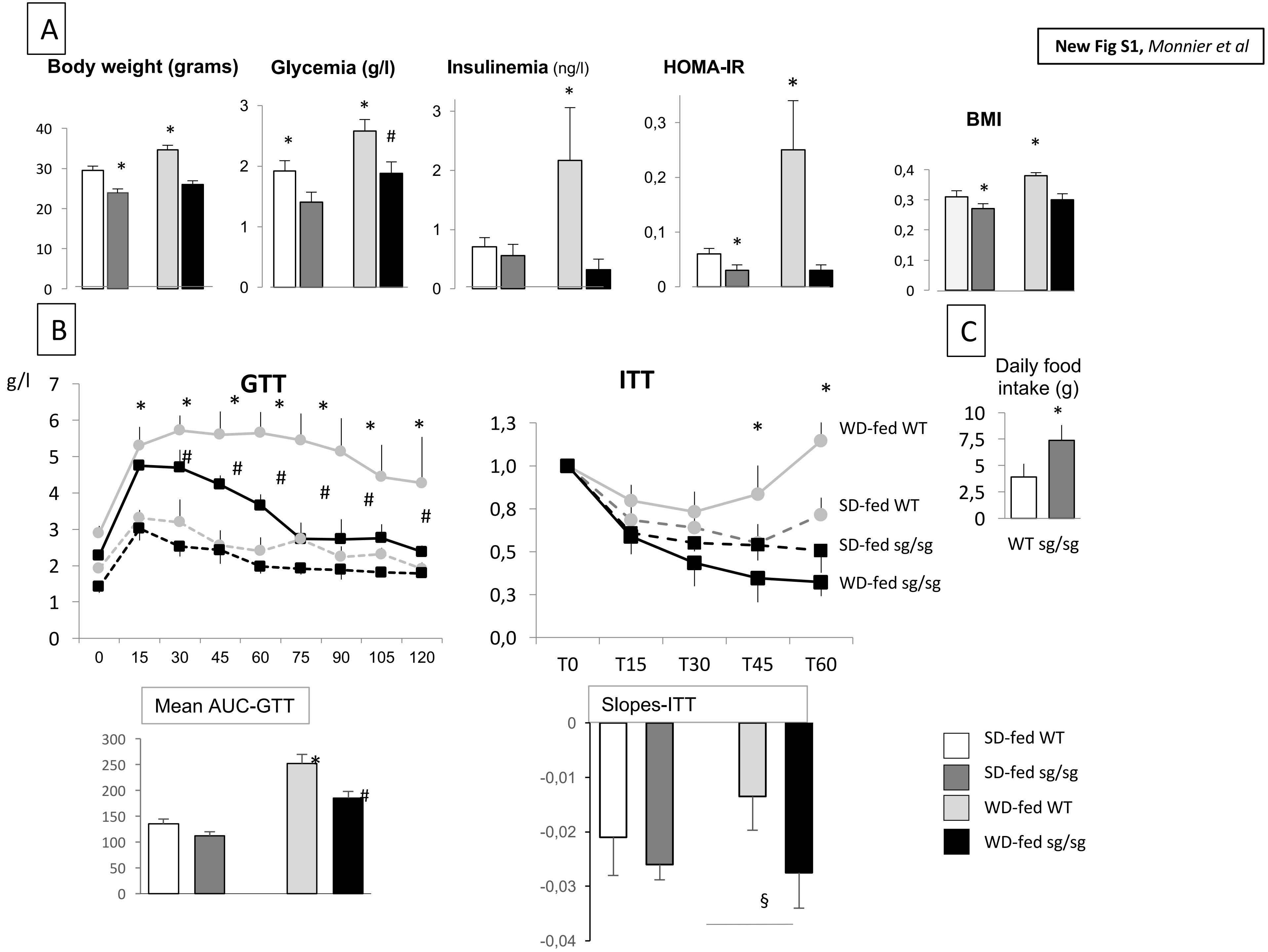

New Fig S1, Monnier et al
A
Body weight (grams)
Glycemia (g/l)
Insulinemia (ng/l)
HOMA-IR
BMI
B
GTT
g/l
ITT
WD-fed WT
SD-fed WT
SD-fed sg/sg
WD-fed sg/sg
T0
T15
T30
T45
T60
Mean AUC-GTT
Slopes-ITT
C
Daily food intake (g)
WT sg/sg
SD-fed WT
SD-fed sg/sg
WD-fed WT
WD-fed sg/sg
