# Supplementary figures and images for: The nuclear retinoid‐related orphan receptor ROR α controls circadian thermogenic programming in white fat depots
Source: Physiol Rep. 2018 Apr 19;6(8):e13678. doi: 10.14814/phy2.13678 (PMC5907938; doi:10.14814/phy2.13678)

**A**

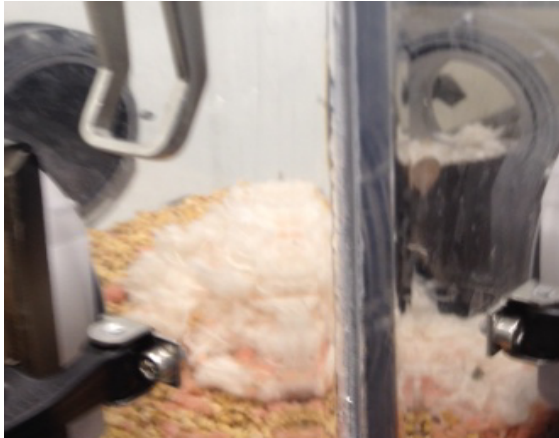

Male WT

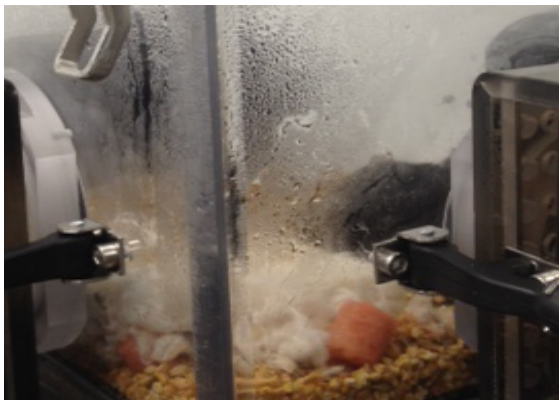

Male sg/sg

**B**

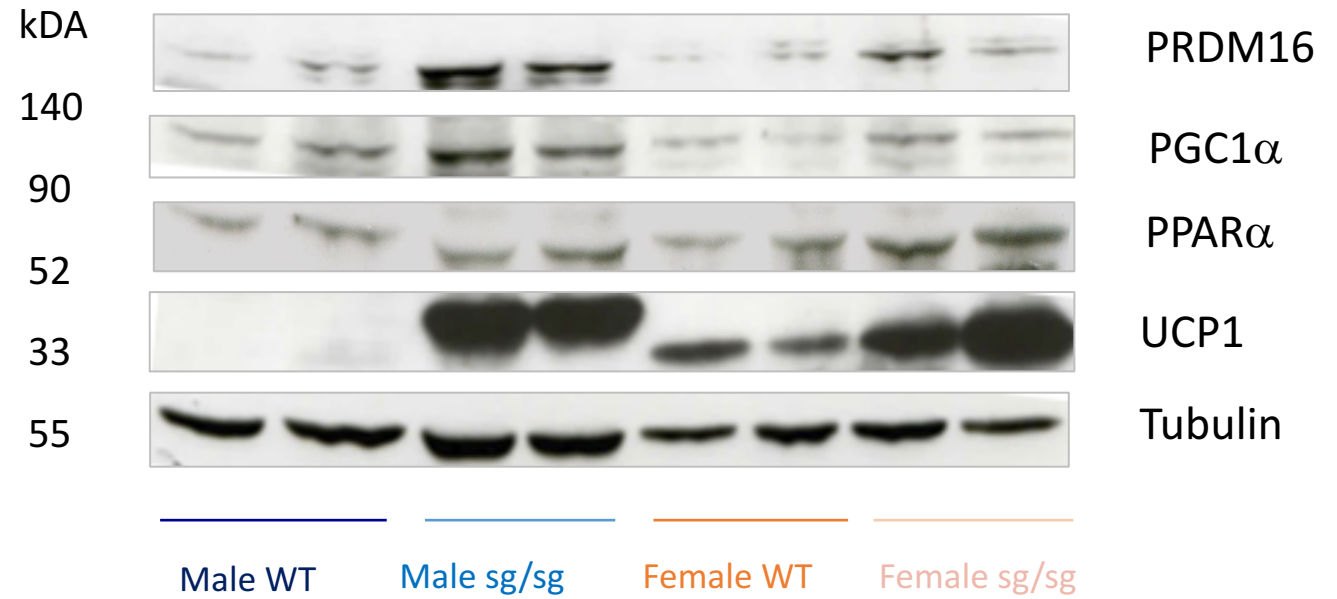

**UCP1 / tubuline**

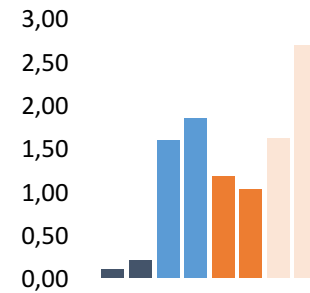

**PRDM16/ tubuline**

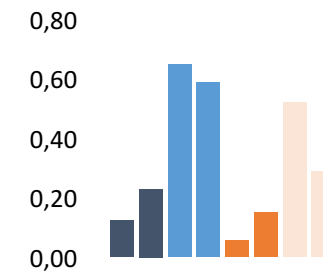

Supplement: Supplementary file 2 — Figure S2: WD‐induced thermogenesis in sg/sg mice. [file PHY2-6-e13678-s002.pdf]

IAT

Female WT

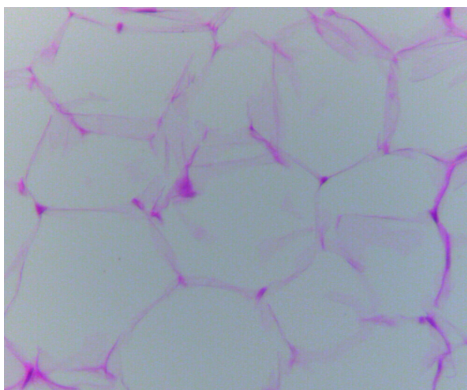

Female sg/sg

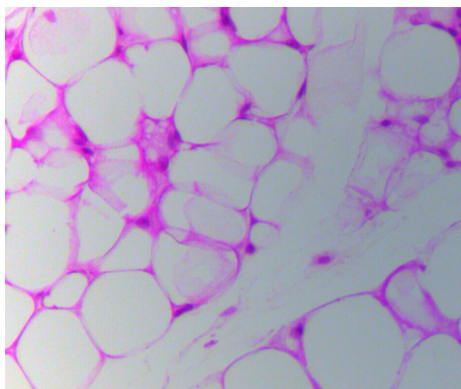

Female WD-sg/sg

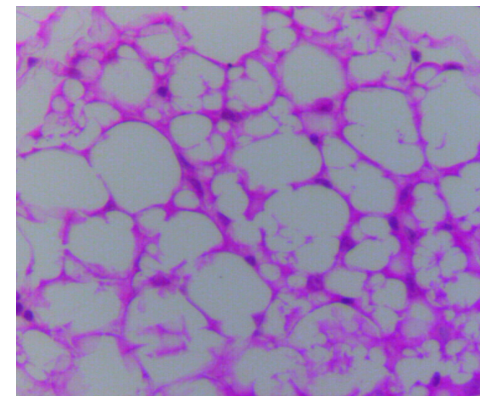

New Fig S3, Monnier et al

PGAT

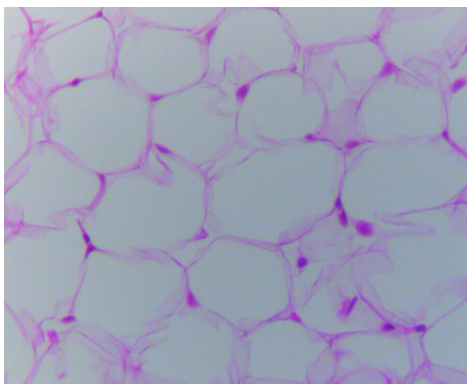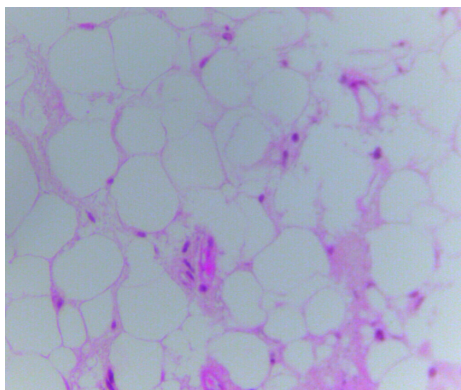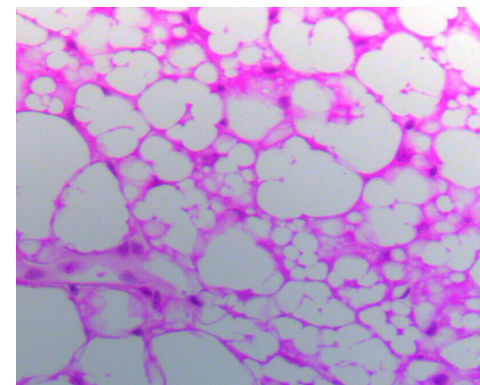

BAT

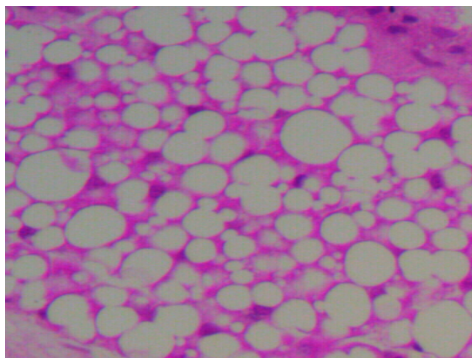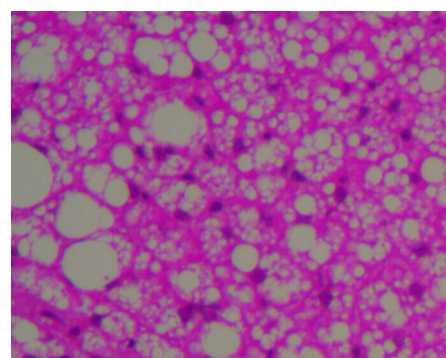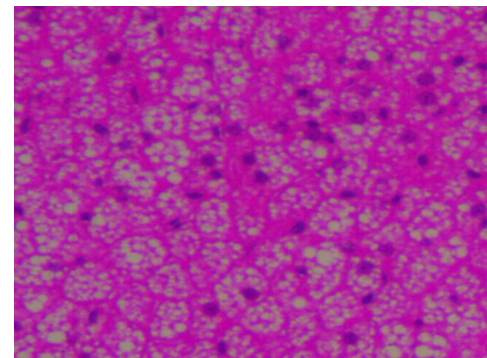

Supplement: Supplementary file 3 — Figure S3: Histology of inguinal (IAT), perigonadal (PGAT), and brown (BAT) adipose tissue of 26‐week‐old female WT and sg/sg mice fed standard or Western diet. [file PHY2-6-e13678-s003.pdf]

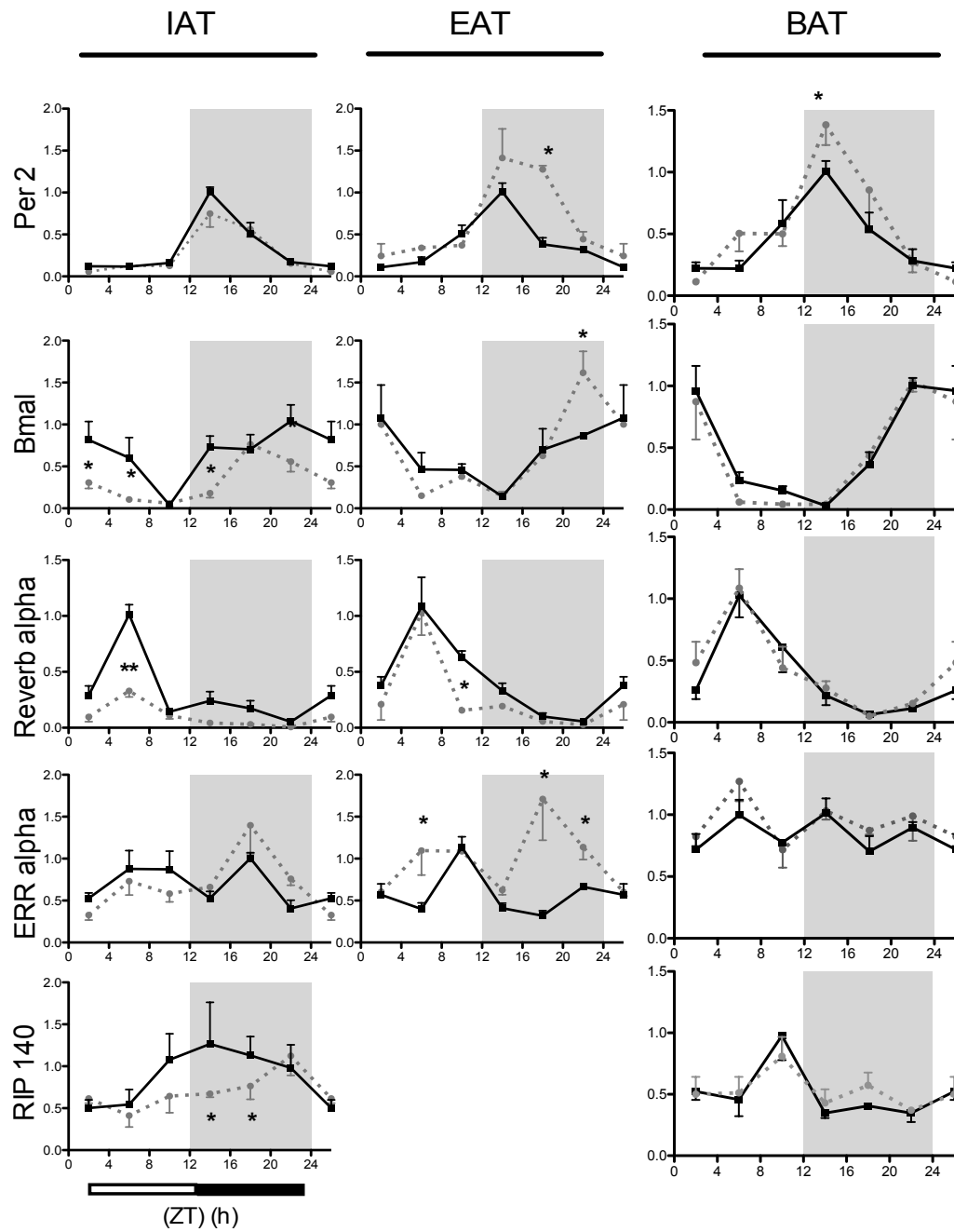

Supplement: Supplementary file 4 — Figure S4: Circadian pattern of some genes expression into the two WAT depots and BAT of sg/sg and WT mice. [file PHY2-6-e13678-s004.pdf]
